# Association of the Telomerase Reverse Transcriptase rs10069690 Polymorphism with the Risk, Age at Onset and Prognosis of Triple Negative Breast Cancer

**DOI:** 10.3390/ijms24031825

**Published:** 2023-01-17

**Authors:** Karin Zins, Elisabeth Peka, Heidi Miedl, Stefanie Ecker, Dietmar Abraham, Martin Schreiber

**Affiliations:** 1 Division of Cell and Developmental Biology, Center for Anatomy and Cell Biology, Medical University of Vienna, A-1090 Vienna, Austria; 2 Department of Obstetrics and Gynecology, Medical University of Vienna, A-1090 Vienna, Austria; 3 Comprehensive Cancer Center (CCC), Medical University of Vienna, A-1090 Vienna, Austria

**Keywords:** breast cancer, telomerase reverse transcriptase (TERT), TERT expression, single nucleotide variant (SNV) rs10069690, breast cancer risk, breast cancer prognosis, molecular subtypes, triple-negative breast cancer (TNBC)

## Abstract

Telomerase reverse transcriptase (TERT) plays a key role in the maintenance of telomere DNA length. The rs10069690 single nucleotide variant, located in intron 4 of TERT, was found to be associated with telomere length and the risk of estrogen receptor-negative but not–positive breast cancer. This study aimed at analysis of the association of rs10069690 genotype and TERT expression with the risk, age at onset, prognosis, and clinically and molecularly relevant subtypes of breast cancer. Accordingly, rs10069690 was genotyped in a hospital-based case-control study of 403 female breast cancer patients and 246 female controls of a Central European (Austrian) study population, and the mRNA levels of TERT were quantified in 106 primary breast tumors using qRT-PCR. We found that in triple-negative breast cancer patients, the minor rs10069690 TT genotype tended to be associated with an increased breast cancer risk (OR, 1.87; 95% CI, 0.75–4.71; *p* = 0.155) and was significantly associated with 11.7 years younger age at breast cancer onset (*p* = 0.0002), whereas the CC genotype was associated with a poor brain metastasis-free survival (*p* = 0.009). Overall, our data show that the rs10069690 CC genotype and a high TERT expression tended to be associated with each other and with a poor prognosis. Our findings indicate a key role of rs10069690 in triple-negative breast cancer.

## 1. Introduction

Telomeres are terminal nucleoprotein structures of linear chromosomes composed of repetitive DNA sequences ([TTAGGG]n in humans) and bound proteins [1]. These complexes protect chromosome ends from being recognized as DNA double-strand breaks and prevent chromosomes from degradation, end-to-end fusion, atypical recombination, and activation of detrimental DNA damage response pathways [2].

Telomere shortening limits the proliferative potential of cells, which eventually leads to cellular senescence resulting in growth arrest [3]. Escaping replicative senescence is an essential step of oncogenesis, and several mechanisms have been identified that permit tumor cells to extend telomeres and increase their replicative capacity [4]. Thus, the maintenance of telomere length endows tumors with unlimited replicative potential, one of the hallmarks of cancer [5]. Consequently, telomeres are crucial in maintaining chromosome integrity as well as genomic stability [6], and aberrant telomere homeostasis confers cells with replicative immortality ([7] for a recent review).

Telomere length is maintained by the reverse transcriptase telomerase, a ribonucleoprotein enzyme that adds the telomeric repeat sequence directly to the single-strand 3′ overhang to maintain telomere ends that have been shortened during each round of replication [8]. Telomere shortening can be counteracted by activating telomerase [9]. The expression of telomerase is extremely low in differentiated human somatic cells, but reactivation may endow a small population of cells with the ability to survive crisis, at which point they become immortalized [10]. The vast majority of human cancers have been proposed to reactivate telomerase [11,12].

Telomerase is composed of an RNA component (hTR or hTERC) and a catalytic protein, telomerase reverse transcriptase (TERT) [13]. The human TERT gene is located on the short (p) arm of chromosome 5 at position 15.33 (5p15.33) and plays a decisive role in the maintenance of telomere DNA length. The TERT gene is highly conserved with low genetic diversity at this locus [14,15], suggesting that subtle variation at the TERT locus may have a disproportionally large effect on telomere biology [16]. Accordingly, mutations in the coding region of TERT can affect telomerase activity and telomere length and generate severe clinical phenotypes, including increased cancer frequency [16].

Genome-wide association studies (GWAS) have demonstrated that single nucleotide variants (SNVs) at chromosome 5p15.33, which contains the TERT gene, are significantly associated with cancer risk [17,18,19,20]. Different studies have suggested that the rs10069690 variant in the TERT gene is a risk factor for several types of cancer, including breast cancer [17,18,21,22,23] and ovarian, lung, and thyroid cancer [24]. rs10069690 is associated with an increased risk of ER-negative, but not ER-positive breast cancer [18,25,26]. A previous study showed that the minor allele of rs10069690 creates an additional splice site in intron 4 of hTERT, causing the production of an alternatively spliced INS1b transcript resulting in a decrease in telomerase activity [27].

These results led us to analyze the association of the intronic rs10069690 SNV in the human TERT gene in a hospital-based case-control study of 403 breast cancer patients and 246 female controls. We found that the age at onset of patients with triple-negative breast cancer (TNBC) with the TERT rs10069690 TT genotype was significantly younger than of those with the CC genotype. On the other hand, the rs10069690 CC genotype tended to be associated with a poor prognosis and was significantly associated with poor overall survival (OS) in ER-positive patients. The CC genotype also tended to be associated with poor overall survival and metastasis-free survival in patients with a mutated TP53 in their tumors. We observed a highly significant association of the CC genotype with poor brain metastasis-free survival in triple-negative patients. High TERT expression tended to be associated with poor disease-free survival, particularly in triple-negative patients. Our results show a subtype-specific association of rs10069690 genotypes with breast cancer risk and prognosis in the investigated Austrian population.

## 2. Results

### 2.1. The TERT rs10069690 SNV and Breast Cancer Risk

A single nucleotide variant (SNV; SNP) located in intron 4 of the human TERT gene (rs10069690; c.1951-205G>A; g.20373G>A; g.1279675C>T; hereafter referred to as rs10069690, with alleles C and T) was genotyped in a hospital-based case-control study of 403 breast cancer patients and 246 female controls. Clinical characteristics of the study population, together with the frequency of the rs10069690 genotypes in the study population and its subpopulations, are shown in Table S1. The control population (*p* = 0.92) and the patient population (*p* = 0.81) were both in Hardy-Weinberg equilibrium. The frequencies of the genotypes CC, CT, and TT were 0.504, 0.417, and 0.079 in patients and 0.5, 0.415, and 0.085 in controls (Table S1). The frequency of the minor T-allele (MAF) was 0.288 in patients and 0.293 in controls, close to the MAF of 0.26 reported for Europeans by the NCBI allele frequency aggregator [28]. To assess the breast cancer risk associated with rs10069690, crude and adjusted odds ratios (OR), 95% confidence intervals (CI), and *p*-values were determined for rs10069690 genotypes and alleles (Table 1). This analysis revealed odds ratios close to one (1; unity), and none of the genotypes or alleles of rs10069690 was associated with a significantly increased or decreased breast cancer risk (Table 1).

### 2.2. Exploratory Analysis of rs10069690 and Breast Cancer Risk in Subpopulations

We next examined potential associations of rs10069690 with breast cancer risk in clinically and histopathologically relevant subpopulations and determined per-allele ORs (T vs. C) as well as ORs for homozygous comparisons (TT vs. CC; Table 2). However, none of the associations in our exploratory analysis were significant at the *p* < 0.05 level (Table 2). rs10069690 has been found to be associated with an increased risk of ER-negative and triple-negative breast cancer [18,26]. We found the following ORs in ER-negative patients: TT vs. CC, OR, 1.63; 95% CI, 0.78–3.40; *p* = 0.208 and T vs. C, OR, 1.20; 95% CI, 0.86–1.87; *p* = 0.275. In patients with triple-negative tumors, our results were: TT vs. CC, OR, 1.87; 95% CI, 0.75–4.71; *p* = 0.155 and T vs. C, OR, 1.35; 95% CI, 0.88–2.05; *p* = 0.167 (Table 2).

### 2.3. Association of the TERT rs10069690 SNV with the Age at Breast Cancer Onset

**Figure 1:**
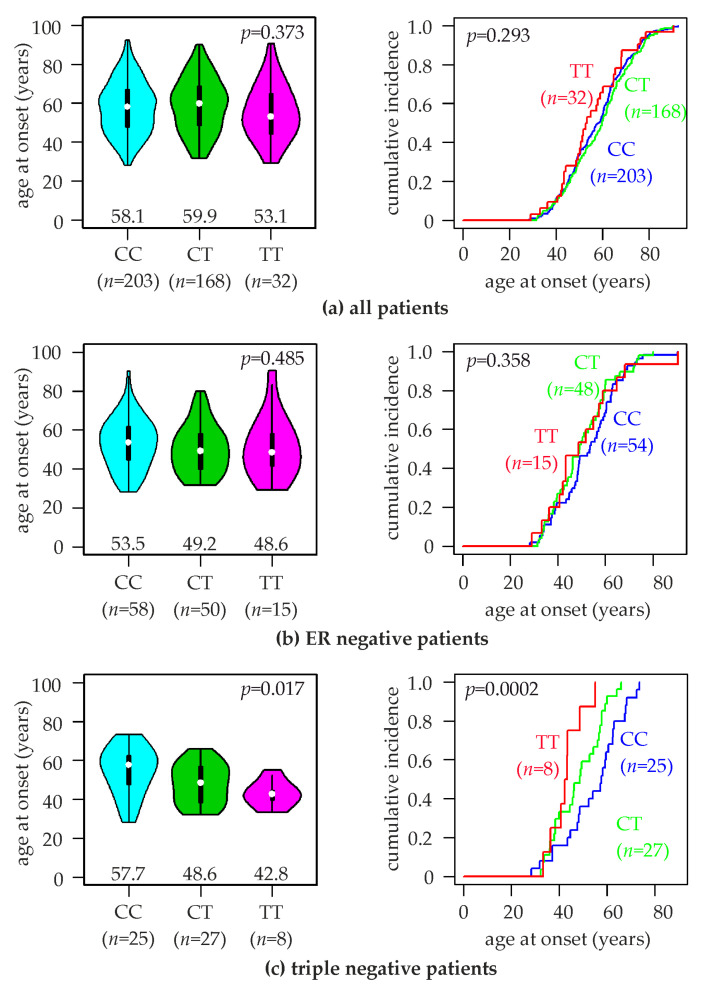
Association of rs10069690 genotypes with the age of breast cancer onset. Violin plots (left) and curves of the cumulative breast cancer incidence (right) with the indicated age of onset are shown for (**a**) all patients in the study population, (**b**) ER-negative, and (**c**) triple-negative patients. rs10069690 genotypes (CC, CT, TT) and numbers of patients (*n*) are indicated. Numbers in the left panels represent the median age of breast cancer onset for each genotype (indicated by white dots). *p*-values (*p*) in the left panels were calculated with Kruskal-Wallis tests and in the right panels with log-rank tests.

Next, we investigated the potential impact of the rs10069690 SNV on the age at breast cancer onset. We found the following mean ages of breast cancer onset for patients with the three rs10069690 genotypes: CC, 57.7 ±13.5 years (median, 58.1); CT, 58.8 ± 13.8 years (median, 59.9); TT, 55.5 ± 14.0 years (median, 53.1; *p* = 0.37, Kruskal-Wallis test; Figure 1a). Thus, patients with the TT genotype exhibited a 2.7 years younger mean age at onset than patients with the CC + CT genotypes (55.5 vs. 58.2 years, *p* = 0.39). Since rs10069690 was found to be strongly associated with the risk of ER-negative and triple-negative breast cancer [25,26] (see also Table 2), we repeated this analysis in these two subtypes. In ER-negative patients, the mean age at breast cancer onset was considerably younger than in unselected patients: CC, 52.9 ±13.0 years (median, 53.5); CT, 50.3 ± 12.5 years (median, 49.2); TT, 50.9 ± 15.8 years (median, 48.6; *p* = 0.5; Figure 1b). Accordingly, ER-negative patients with the CC genotype exhibited a 2.5 years older mean age at onset than those with the CT + TT genotypes (52.9 vs. 50.4 years, *p* = 0.9). In triple-negative cases (i.e., negative for estrogen receptor, progesterone receptor, and HER2), a significant association of the mean age at onset with rs10069690 genotype was observed: CC, 54.5 ±12.4 years (median, 57.7); CT, 48.0 ± 10.1 years (median, 48.6); TT, 42.9 ± 6.8 years (median, 42.8; *p* = 0.017, Kruskal-Wallis test; *p* < 0.0002, log-rank test; Figure 1c). Thus, the mean age at the onset of triple-negative patients with the TT genotype was 11.7 years younger than those with the CC genotype (*p* = 0.043). Triple-negative patients with the CT genotype had an intermediate age at onset (Figure 1c).

### 2.4. Association of TERT Expression in Primary Breast Tumors with Clinical and Histopathological Patient Characteristics

**Figure 2:**
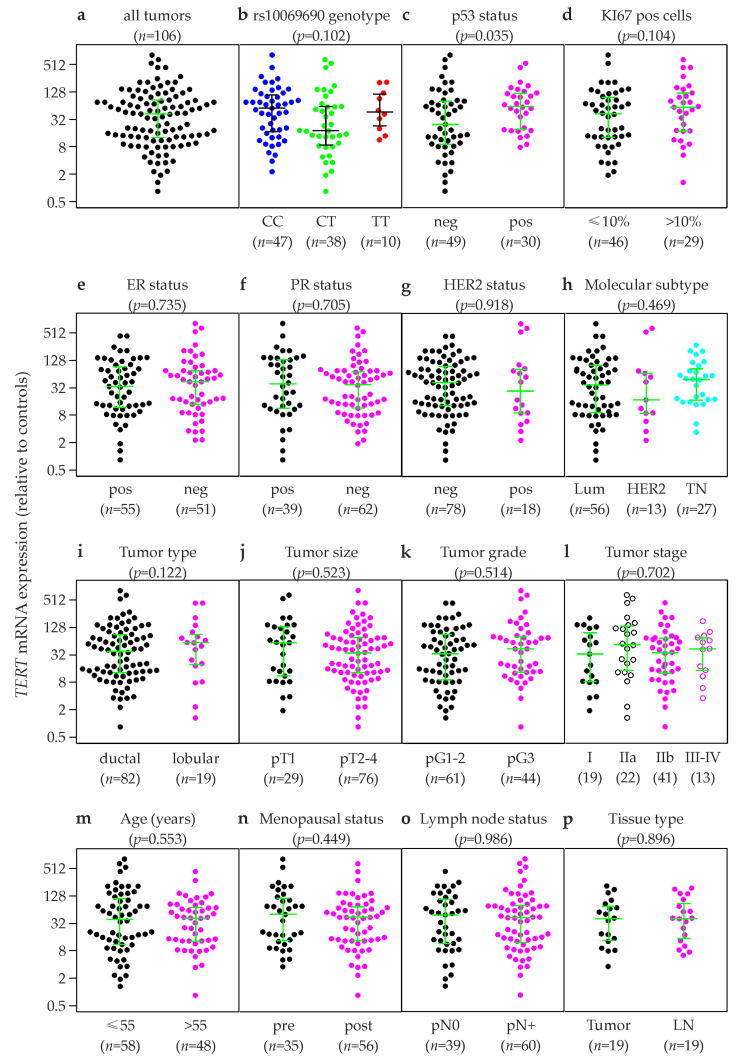
Association of *TERT* mRNA expression with established clinical and histopathological parameters. Strip charts of *TERT* expression of all tumors (**a**), and according to rs10069690 genotype (**b**), p53 status (**c**), KI67 status (**d**; ≤10% vs. >10% KI67 pos cells), estrogen receptor (ER) status (**e**), progesterone receptor (PR) status (**f**), HER2-status (**g**), molecular subtype [**h**; luminal A and B (Lum), HER2-type (HER2), and triple-negative (TN)], tumor type (**i**), size (**j**), grade (**k**) and stage (**l**); age at breast cancer onset (**m**), menopausal status (**n**), lymph node status (**o**), and in paired primary tumors and lymph node metastases (LN; **p**). neg, negative; pos, positive. The numbers of patients in each group (*n*) are shown in parentheses. The y-axes show normalized relative *TERT* mRNA levels (linear values). Horizontal lines in panels indicate the first, second (i.e., median), and third quartiles. *p*-values (*p*, in parentheses above each panel) were determined by Kruskal-Wallis tests.

Relative *TERT* mRNA expression levels were successfully quantified by qRT-PCR in primary tumor tissue samples of 106 breast cancer patients diagnosed between 1989 and 1993. All tumors but one exhibited considerably elevated *TERT* expression levels compared to four normal breast tissue controls (mean, 32.5-fold; median, 41.5-fold; range, 0.83 to 813.4-fold; Figure 2a), consistent with the finding that *TERT* expression is switched off in differentiated adult tissues, but reactivated in most tumors [29]. Moreover, two largely non-overlapping groups among these 106 breast tumors emerged from the hourglass shape of the strip charts (Figure 2a), one with moderate TERT overexpression (up to ≈25-fold compared to controls; *n* = 49; 46%) and the other with high-level overexpression (≈32- to 810-fold compared to controls; *n* = 57; 54%). Incidentally, the cutoff between these two groups is almost identical to the mean expression level of the entire population (*n* = 106).

To investigate which biological or clinical factors influence this bimodal expression of TERT, associations of *TERT* expression with well-established clinical and histopathological characteristics of breast cancer were visualized with strip charts (Figure 2). Using the mean as a cutoff, 46% of the tumors (49/106) were moderate overexpressors, and 54% were high-level overexpressors (54/106). In contrast, 70% of the tumors with the rs10069690 TT genotype were high-level overexpressors (7/10), whereas only 42% of CT tumors were high-level overexpressors (16/38). However, the mean *TERT* expression levels were not significantly different in tumor samples of patients carrying any of the three rs10069690 genotypes (*p* = 0.102; Kruskal-Wallis test; Figure 2b). Mean expression levels were 1.8-fold lower in CT vs. CC tumors (*p* = 0.048), but no additional significant differences between genotypes were observed.

Moreover, 70% of the tumors of patients with a positive p53 status were high-level overexpressors (21/30), and overall mean *TERT* expression levels were elevated in these tumors (2.1-fold; *p* = 0.035; Figure 2c). 66% of the tumors with >10% KI67 positive cells were high-level overexpressors (19/29; 1.5-fold overall upregulation; *p* = 0.104; Figure 2d). 59% of triple negative tumors were high-level overexpressors (16/27), and their mean TERT expression was 1.3-fold higher than in luminal tumors (*p* = 0.379) and 1.5-fold higher than in HER2-type tumors (*p* = 0.231). Moreover, 63% of lobular tumors were high-level overexpressors (12/19), and their mean *TERT* expression was 1.2-fold upregulated (*p* = 0.122; median, 1.5-fold up; Figure 2i). No significant association with *TERT* expression was found for ER-, PR- and HER2 status, tumor size, -grade and -stage, age, menopausal status, and lymph node status (Figure 2).

### 2.5. TERT Expression and rs10069690 Genotype in Breast Cancer Cell Lines

**Figure 3:**
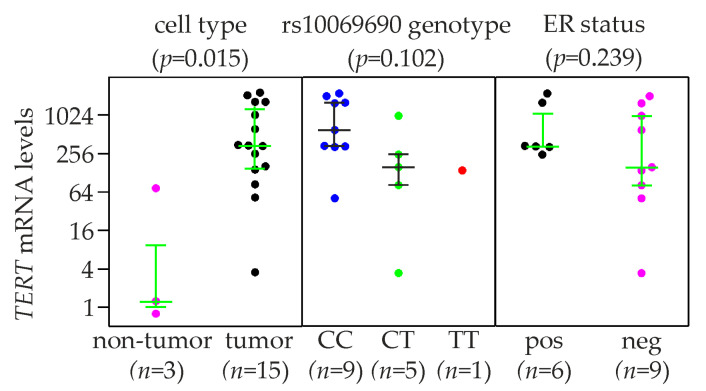
Expression of *TERT* in 19 cell lines derived from breast tumors and untransformed breast epithelium. Cell lines were stratified by cell type (untransformed mammary epithelial vs. breast cancer cell lines), rs10069690 genotype, and estrogen receptor (ER) status as indicated (the latter two in breast cancer cell lines only). MCF-10A had by far the highest *TERT* mRNA levels among the non-tumor cell lines, and Hs 578T by far the lowest among the breast cancer cell lines (Table S2). The y-axes show normalized relative *TERT* mRNA levels (linear values). The numbers of cell lines in each group (*n*) are shown in parentheses. Horizontal lines indicate the first, second (i.e., median), and third quartiles. Pos, positive; neg, negative. ER status according to [30,31]. *p*-values (*p*, in parentheses above each panel) were determined by Kruskal-Wallis tests.

Relative TERT mRNA expression levels were determined in breast cancer (*n* = 15) and normal (non-tumor) breast cell lines (Hs 578Bst, MCF-10A, MCF-10F, and HMEC; *n* = 4). Two non-tumor cell lines (HMEC and MCF-10F) expressed TERT at very similar levels, whereas expression in Hs 578Bst was below detection, and MCF-10A expressed TERT at a ≈70-fold higher level than the average of HMEC and MCF-10F (Figure 3; Table S2). The 15 breast cancer cell lines expressed TERT at ≈75-fold higher mean levels than the three non-tumor cell lines (median, ≈275-fold; *p* = 0.015, Kruskal-Wallis test). Similar to normal cells, there was one outlier among the breast cancer cell lines, Hs 578T, which expressed TERT at ≈125–fold lower levels than the mean of the remaining 14 cell lines. As in breast tumors, mean TERT expression was higher in breast cancer cell lines with the rs10069690 CC genotype (*n* = 9) than in those with the CT/TT (*n* = 5 + 1) genotype (5.7–fold; *p* = 0.102, Kruskal-Wallis test of all three genotypes; Figure 3). ER-positive breast cancer cell lines exhibited 2.8-fold higher mean TERT mRNA levels than ER-negative ones (Figure 3). However, due to the high variability, these differences were not significant (*p* = 0.239).

### 2.6. Association of rs10069690 Genotype with Breast Cancer Prognosis

**Figure 4:**
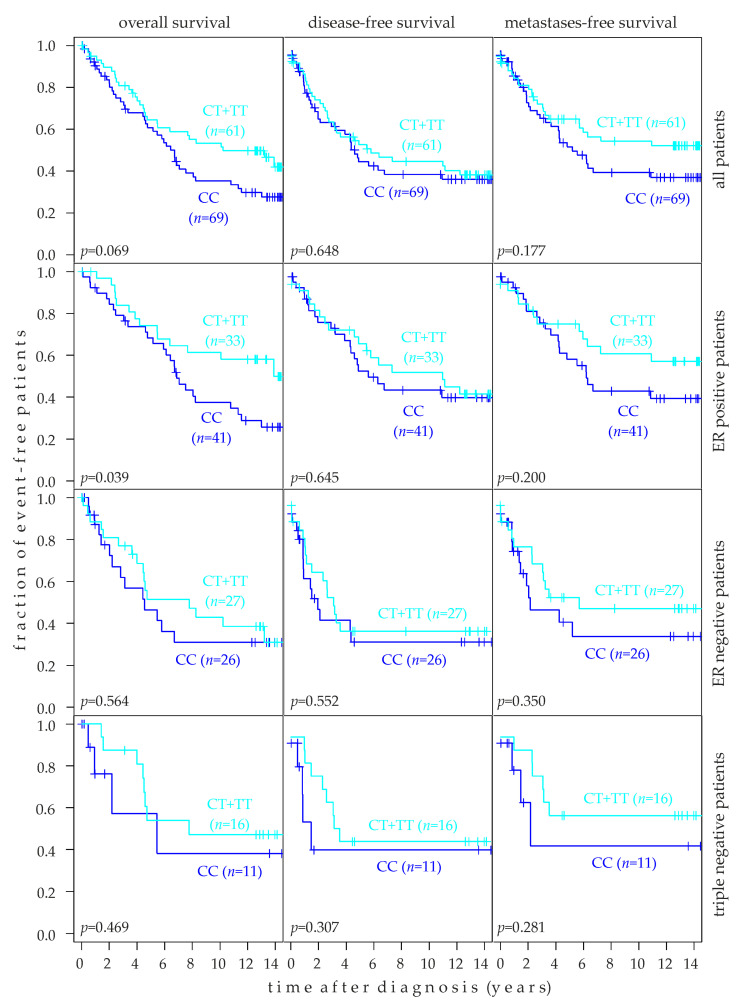
Association of rs10069690 genotypes with the survival of human breast cancer patients. Patients with CT and TT genotypes were combined into one group and compared to CC patients. Kaplan-Meier analyses of the overall survival, disease-free survival, and metastasis-free survival in unselected patients (top row, *n* = 130), estrogen receptor (ER) positive patients (second row; *n* = 74), ER-negative patients (third row; *n* = 53), and triple-negative patients (bottom row; *n* = 27) are shown. Numbers (*n*) of patients in each group and *p*-values (*p*) are indicated.

The association of rs10069690 genotypes with the overall survival (OS), disease-free survival (DFS), and metastasis-free survival (MFS) was assessed in Kaplan-Meier analysis of 130 patients and in subsets thereof. Since the number of patients with the TT genotype was small (*n* = 10 in the entire population; *n* = 6 in estrogen receptor (ER) negative patients; *n* = 4 in ER-positive and in triple-negative patients), they were grouped together with CT patients, and this group was compared to CC patients (Figure 4). In all patients (*n* = 130), the rs10069690 CC genotype tended to be associated with a poor OS (*p* = 0.069) and MFS (*p* = 0.177; Figure 4, top row). In contrast, no association was observed with the DFS (*p* = 0.648). Interestingly, the CC genotype was also associated with a 1.6-fold higher TERT expression than CT + TT in breast tumors (see Section 2.4). This analysis was also performed in ER-positive (*n* = 74), ER-negative (*n* = 53), and triple-negative patients (*n* = 27). The rs10069690 CC genotype was significantly associated with poor OS in ER-positive patients (*p* = 0.039). Although the CC genotype tended to be associated with a poor prognosis in all other analyses as well, no further associations were significant at the *p* < 0.05 level (Figure 4). Moreover, the CC genotype also tended to be associated with a poor OS (*p* = 0.100) and MFS (*p* = 0.165) in patients with mutated TP53 in their tumors (Figure S1).

### 2.7. Association of TERT Expression with Breast Cancer Prognosis

**Figure 5:**
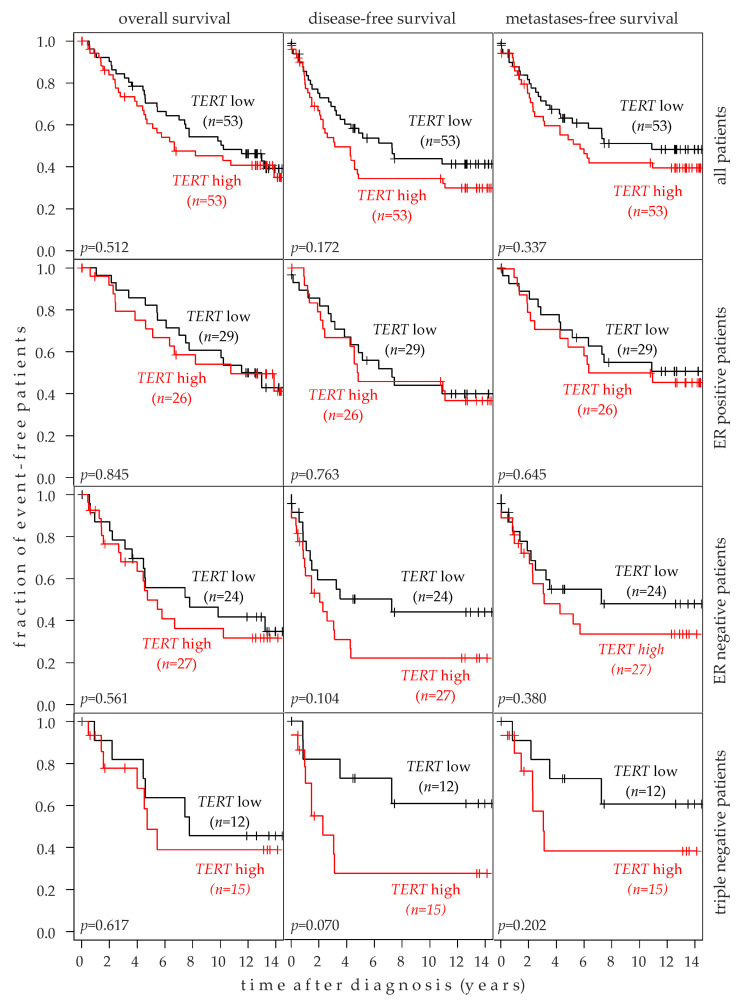
Association of *TERT* mRNA expression with the survival of human breast cancer patients. Kaplan-Meier analyses of the overall survival, disease-free survival, and metastasis-free survival in unselected patients (top row, *n* = 106), estrogen receptor (ER) positive patients (second row; *n* = 55), ER-negative patients (third row; *n* = 51), and triple-negative patients (bottom row; *n* = 27) are shown. *TERT* high, *TERT* expression above the median of the study population (*n* = 106); *TERT* low, *TERT* expression below the median.

The association of TERT expression with the OS, DFS, and MFS was assessed in Kaplan-Meier analysis of 106 patients and in subsets thereof. In all analyses, a high TERT expression tended to be associated with poor survival (Figure 5). In contrast to the rs10069690 genotype, the closest association with TERT expression was observed for the DFS, particularly in triple-negative patients (*p* = 0.07) and ER-negative patients (*p* = 0.104). Conversely, no association of TERT expression with the OS, DFS, or MFS was observed in ER-positive patients (Figure 5). Likewise, no association was observed in patients with wild type or with a mutated TP53 gene in their tumors (Figure S1).

### 2.8. Association of rs10069690 Genotype with Target Tissue Specific Metastasis-Free Survival

**Figure 6:**
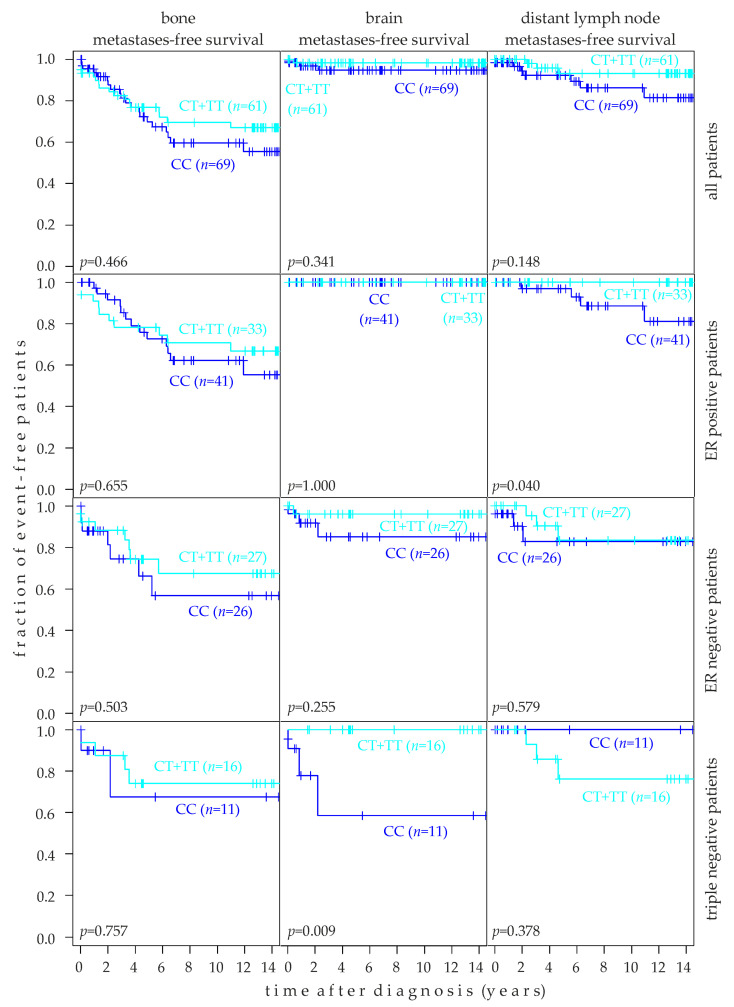
Association of rs10069690 genotypes with the target tissue-specific metastasis-free survival of human breast cancer patients. Patients with CT and TT genotypes were combined into one group and compared to CC patients. Kaplan-Meier analyses of the bone metastasis-free survival, brain metastasis-free survival, and survival free of metastasis to distant lymph nodes in unselected patients (top row, *n* = 130), estrogen receptor (ER) positive patients (second row; *n* = 74), ER-negative patients (third row; *n* = 53), and triple-negative patients (bottom row; *n* = 27) are shown. Numbers (*n*) of patients in each group and *p*-values (*p*) are indicated.

The association of rs10069690 genotypes with the bone metastasis-free survival, brain metastasis-free survival, and survival free of metastasis to distant (i.e., non-locoregional) lymph nodes (dLN) was assessed in Kaplan-Meier analysis of 130 patients, and in subsets thereof. Patients with the CT and TT genotypes were again grouped together, and this group was compared to CC patients (Figure 6). In all patients (*n* = 130), the rs10069690 CC genotype tended to be associated with a poor survival free of metastases to bone (*p* = 0.466), brain (*p* = 0.341), and distant lymph nodes (*p* = 0.148; Figure 6). The CC genotype was significantly associated with poor dLN metastasis-free survival in ER-positive patients (*p* = 0.04). All dLN metastases in ER-positive patients occurred in CC patients only (Figure 6). rs10069690 was not associated with bone (*p* = 0.655) or brain (*p* = 1) metastasis-free survival in ER positive patients. No brain metastases were observed in ER-positive patients (*n* = 74) in our study, irrespective of the rs10069690 genotype (Figure 6). Likewise, the rs10069690 genotype was not associated with MFS in any of the three target tissues studied in ER-negative patients (Figure 6). In contrast, the association of the CC genotype with poor brain metastasis-free survival was highly significant in triple negative patients (*p* = 0.009); no brain metastases occurred in triple negative CT or TT patients (*n* = 16; Figure 6). Of note, dLN metastasis-free survival in triple-negative patients was our only analysis in which the CC genotype tended to be associated with a good prognosis, albeit non-significantly (*p* = 0.378; Figure 6). Analogous analyses revealed no significant association of TERT expression with the bone-, brain-, or dLN metastasis-free survival (Figure S2). 

## 3. Discussion

The TERT gene encodes the catalytic subunit of telomerase, which controls telomere length, a process linked with genomic instability and implicated in cellular immortalization and malignant transformation [6]. Given the fundamental role of TERT in oncogenesis, it is not surprising that variants within the TERT gene have been associated with increased cancer risk [16]. A number of studies have suggested that the rs10069690 polymorphism, located in intron 4 of the TERT gene, is a risk factor for several types of cancer, including breast cancer [24,25]. Moreover, the risk-associated minor allele (T) of rs10069690 was shown to result in a TERT mRNA splice variant, which reduces overall telomerase activity. This, in turn, may increase the risk of short telomeres in normal adult tissues, which can increase the risk of genetic instability and predispose to cancer.

Breast cancer is a heterogeneous disease; different molecular subtypes are associated with distinct biology, prognosis, and potential for therapy. In the present study, we found differences in the strength of the association of rs10069690 with breast cancer risk in clinically relevant subpopulations. Stratification by estrogen receptor (ER) status showed elevated ORs in ER-negative (per-allele OR, 1.20; 95% CI, 0.86–1.67; *p* = 0.275) but not in ER-positive tumors, in agreement with previous studies [18]. ORs were also elevated in tumors with a high fraction of KI67-positive tumor cells (per-allele OR, 1.56; 95% CI, 0.88–2.75; *p* = 0.133). Likewise, stratification by molecular subtype revealed the lowest odds ratios in luminal tumors and the highest in triple-negative tumors (per-allele OR, 1.35; 95% CI, 0.88–2.05; *p* = 0.167). Remarkably, we found that the mean age at onset of triple-negative patients with the TT genotype was 11.7 years younger than of those with the CC genotype. Moreover, the strength of association of high TERT expression with a poor prognosis, particularly the DSF, was higher in triple negatives (*p* = 0.07) than in other subtypes, and the rs10069690 CC genotype was significantly associated with a poor brain metastasis-free survival in triple-negative patients (*p* = 0.009), but not in other subtypes. Collectively, these data indicate that the rs10069690 SNV and TERT expression play a central role in triple-negative breast cancer (TNBC). Interestingly, compared to women of European ancestry (risk allele frequency of 26%), the frequency of the risk allele of rs10069690 is substantially higher in African American women (57%), a population in which also ER-negative and triple-negative breast cancer are more frequent [18]. Based on this finding, it has been suggested that the rs10069690 locus may be responsible for an up to 15% higher incidence rate of ER-negative or triple-negative breast cancer in women of African compared to European ancestry, highlighting population-dependent differences [18].

In our study, we observed an association of the rs10069690 CC genotype with poor overall and metastasis-free survival of different breast cancer subpopulations. We showed that the rs10069690 CC genotype was significantly associated with poor overall survival (*p* = 0.039) and dLN metastasis-free survival of ER-positive patients (*p* = 0.040) and with a poor brain metastasis-free survival of triple-negative patients (*p* = 0.009). Although the CC genotype tended to be associated with a poor prognosis in all other analyses as well, all other associations were not significant at the *p* < 0.05 level. Since the exact biological function of rs10069690 is not yet clear [24], we can only speculate why the common CC genotype, as opposed to the minor TT genotype, is associated with a poor prognosis. Decreased telomerase activity has been previously demonstrated for the T-allele of rs10069690 [27]. This decreased telomerase activity could be a rate-limiting factor in cancer cell proliferation and cancer progression and could thus make cancer cells with the TT genotype less aggressive and hence associated with a favorable prognosis [32]. Consistently, high TERT expression (and hence a higher telomerase activity) tended to be associated with poor disease-free survival in the present study, particularly in triple-negative patients (*p* = 0.07) and in ER-negative patients (*p* = 0.104).

There is little information on the association of the rs10069690 genotype with target tissue-specific metastasis-free survival in breast cancer patients. Our data show very clear differences in the distribution of genotypes, with triple-negative patients standing out in particular. We found a highly significant association of the CC genotype with poor brain metastasis-free survival in triple-negative patients (*p* = 0.009). No brain metastases occurred in triple-negative CT or TT patients. In contrast, the rs10069690 genotype was not associated with MFS in any of the three target tissues studied in ER-negative patients. These findings are in line with data showing that the breast cancer molecular subtype strongly impacts the occurrence and kinetics of brain metastases and the prognosis of the patients [33]. In this study, hormone receptor-negative, HER2-positive, and triple-negative tumors had a higher risk of developing brain metastases [33].

In contrast, the CC genotype was significantly associated with poor distant lymph node (dLN) metastasis-free survival in ER-positive patients (*p* = 0.04). All dLN metastases in ER-positive patients occurred in CC patients only. rs10069690 was not associated with bone (*p* = 0.655) or brain metastasis-free survival (*p* = 1) in ER positive patients. These data again demonstrate a molecular subtype-dependent association of the rs10069690 CC genotypes with tissue-specific metastasis. Thus, while the TT genotype is associated with an increased breast cancer risk [18,25,26], the CC genotype is associated with a poor prognosis. Consistently, in breast tumors and cell lines, the CC genotype also tends to be associated with an elevated expression of TERT (*p* = 0.102 in tumors and cell lines), which we and others have found to also be associated with a poor prognosis [34]. However, there was no significant association of TERT expression with bone-, brain-, or dLN metastasis-free survival (*p* = 0.466, *p* = 0.341, and *p* = 0.148, respectively).

In general, however, TERT expression levels were considerably elevated in breast tumors and breast cancer cell lines compared to healthy controls, consistent with the finding that TERT expression is silenced in differentiated adult tissues but reactivated in most tumors [35]. Two largely non-overlapping groups were observed among the 106 breast tumors analyzed, one with moderate TERT overexpression (up to ≈25-fold compared to controls; 46%) and the other with high-level overexpression (≈32- to 810-fold; 54%). However, among the analyzed well-established clinical and histopathological subgroups of breast cancer, there was none that exhibited exclusively moderate or exclusively high-level overexpression. On the other hand, some enrichments of high-level overexpression were observed, e.g., in tumors with the rs10069690 TT genotype (70% of these tumors), in tumors with a positive p53 status (70%), in tumors with >10% KI67 positive cells (66%), and in lobular tumors (63%). Taken together, TERT expression was rather heterogeneous in all subgroups analyzed, and none of these subgroups in isolation can fully explain the observed bimodal expression of TERT. The overall expression of TERT was significantly increased in p53 positive patients, i.e., tumors with a mutation in the TP53 gene (*p* = 0.035) and tended to be increased in tumors with >10% KI67 positive cells (*p* = 0.104). Similar data were found in a recent study, also showing that a high KI67 proliferation index was associated with an increased relative expression of TERT [36]. The TP53 tumor suppressor gene is the most commonly mutated gene in human cancers and functions in many cellular pathways, including regulation of apoptosis, cell cycle control, and DNA damage repair processes [37]. A high fraction of KI67-positive cells and a positive p53 status both indicate a high proliferation rate. Accordingly, these results are biologically plausible, as telomeres shorten with each cell division. Therefore, rapidly proliferating cells probably require increased telomerase activity and TERT expression to compensate [7].

From a prognostic point of view, we have found that high TERT expression tended to be associated with poor survival. In contrast to the rs10069690 genotype, the closest association with TERT expression was observed for the DFS, particularly in triple-negative patients (*p* = 0.07) and in ER-negative patients (*p* = 0.104). In contrast, we found no association between TERT expression and the OS, DFS, or MFS in ER-positive patients or in patients with wild type or with a mutated TP53 gene in their tumors. With respect to survival and TP53 status, the closest association of rs10069690 was seen for overall survival, although not significant, in patients with a mutant TP53 (*p* = 0.100). Expression of TERT and the rs10069690 genotype do not show the same pattern of association in the different subtypes with respect to survival, indicating that additional mechanistic effects of rs10069690 beyond its association with TERT expression play a role here, such as its proposed role in giving rise to an additional splice variant [27].

Our study provides evidence of an association of rs10069690 with breast cancer risk and with tissue-specific breast cancer metastasis in the context of breast cancer subtypes. These results suggest that the role of the rs10069690 variant in carcinogenesis and prognosis is potentially influenced by the molecular subtype and tumor stage. Therefore, we can infer that rs10069690 has subtype-specific contributions and may play different roles in breast cancer subtypes. However, this study showed that, despite some significant associations, there is still a lot of uncertainty about this issue, with conflicting results and rather discrete associations. We consider our subgroup analyses as exploratory (i.e., hypothesis-generating rather than testing of previously formed hypothesis) and therefore did not adjust for multiple testing, as recommended previously [38]. Accordingly, our survival analyses should be interpreted with caution due to the limitations of multiple testing. Future studies with a larger sample size are needed to validate the current results to overcome these limitations. In addition, functional studies are necessary to reveal the role of the TERT rs10069690 genotypes in breast cancer development and progression. Our findings document the molecular and clinical heterogeneity of rs10096960 genotypes within subtypes of breast cancer with respect to risk, age at onset, and prognosis, which is most notable for the triple-negative subtype.

## 4. Materials and Methods

### 4.1. Study Population

Women of European descent and Austrian residency enrolled at the Department of Obstetrics and Gynecology, Medical University of Vienna, were included in this study. Healthy females and consecutive patients with benign gynecological lesions were enrolled as nested controls between 2002 and 2004 (*n* = 255). There were 276 consecutive female breast cancer patients treated between 2002 and 2004, and another 134 consecutive patients treated between 1989 and 1993 enrolled in this study. Malignant breast cancer in all patients was confirmed by histopathology. The clinical and histopathological characteristics of the study population are shown in Table S1. Tumor tissue was isolated prior to the onset of any neoadjuvant or other therapy. In addition to the FFPE tissue samples available from all patients, fresh-frozen tumor tissue was also available from the 134 patients treated between 1989 and 1993, which we used for RNA isolation (see Section 4.4.). Moreover, detailed follow-up records were also available from these 134 patients (the end of the follow-up period was September 2005). Upon completion of genotyping, seven patients and nine control subjects had to be excluded from further analyses due to technical genotyping failure. Accordingly, all analyses shown are based on the remaining 403 breast cancer patients and 246 controls.

### 4.2. Cell Lines

The Research Resource Identifiers (RRIDs) for all cell lines used are provided in Table S2. HMEC (human mammary epithelial cells) were a gift from M. R. Stampfer [39]. All other cell lines were purchased from DSMZ (“Deutsche Sammlung von Mikro-Organismen und Zellkulturen,” Braunschweig, Germany): CAL-51, HCC1143, HCC1937, and KPL-1, or ATCC (American Type Culture Collection, Manassas, USA): AU565, BT-474, CAMA-1, Hs 578T, Hs 578Bst, MCF-7, MCF-10A, MCF-10F, MDA-MB-231, MDA-MB-453, MDA-MB-468, SK-BR-3, T-47D, and ZR-75-1. Cell culture conditions of all cell lines were described previously [40]. DSMZ and ATCC authenticate all cell lines by STR profiling before distribution. Genomic DNA and total RNA were isolated from all cell lines immediately after receipt, i.e., within three to eight passages [40,41].

### 4.3. DNA Isolation and SNV Genotyping

Genomic DNA for genotyping was isolated from EDTA-stabilized blood samples with the QIAamp DNA Blood Midi Kit (Qiagen, Venlo, The Netherlands) and from fresh-frozen tumor tissue with the High Pure PCR Template Preparation Kit (Roche, Vienna, Austria) as described previously [42,43]. DNA was dissolved in TE buffer and stored at −80 °C. SNV rs10069690 was genotyped by TaqMan PCR with Genotyping Master Mix and allele-specific, fluorescently labeled probes (Assay-ID C__30322061_10; Applied Biosystems, Brunn am Gebirge, Austria) on a CFX96 real-time PCR instrument (BioRad, Vienna, Austria). PCR reactions were carried out with 20 ng of genomic DNA in a reaction volume of 10 µL following the manufacturers’ instructions. As quality control measures, (i) two to six negative controls (2 μL ddH_2_O instead of DNA) were included in each qPCR run; (ii) 52 samples were genotyped in duplicate; (iii) Patient, control, and duplicate statues were blinded to the laboratory staff. Genotyping failed for nine controls and seven patients, presumably due to the poor quality of genomic DNA.

### 4.4. qRT-PCR Quantification of mRNA Expression Levels

Primary tumor specimens were obtained at the time of surgery, snap-frozen in liquid nitrogen and stored at −80 °C. Isolation of total RNA from these fresh-frozen tumor samples, quality control, preservation and storage of the isolated RNA, reverse transcription, and quantification of relative mRNA levels of β-actin have been described previously [44,45]. Here, relative TERT mRNA levels were quantified following this previously described procedure, analyzing each sample in duplicate by a quantitative reverse transcription PCR (qRT-PCR; real-time PCR; TaqMan RT-PCR) with a CFX96 real-time PCR instrument (BioRad, Vienna, Austria), using primers and gene-specific fluorescent probes with the following assay-IDs purchased from Applied Biosystems: TERT, hs00972650_m1; β-actin (housekeeping gene control), hs_99999903_m1. Patient characteristics were blinded to the laboratory staff. In each qRT-PCR run, two to four negative controls (2.5 μL ddH_2_O instead of cDNA) were included. No signal was detected in any of these reactions. As a positive control, duplicate samples of serial dilutions of a cDNA standard were included in each run. In order to derive relative mRNA levels, the Ct (threshold cycle) values of TERT were normalized to those of β-actin in each sample, producing ΔCt values: ΔCt*_TERT_* = mean (Ct*_TERT_*-Ct_ß-actin_). ΔCt values were further normalized to control by expressing the expression levels of all tumor samples relative to the mean of four control RNA samples from normal breast tissue purchased from commercial suppliers, producing ΔΔCt values. Two non-cancer cell lines (HMEC and MCF-10F; see Section 4.2) were used as normalization controls for breast cancer cell lines. All relative mRNA expression levels are presented as 2^−ΔΔCt^ values (i.e., as linear values, but on a log [2] ordinate) as described [44,45]. Quantification of TERT mRNA failed for 5 out of 111 tumor samples, presumably due to poor RNA quality.

### 4.5. Statistical Analyses

Statistical analyses were performed with R 3.3.2, an open-source language and environment for statistical computing, available from www.r-project.org (accessed on 24 October 2022) [46]. Rs10069690 genotype is a categorical variable with three categories/genotypes and was handled as such. For some analyses, two genotypes were combined into one category, and were compared to the third genotype as second category (e.g., CT + TT vs. CC). TERT expression is a continuous variable and was handled as such wherever possible. In all these analyses, the normalized log [2] values, i.e., ΔΔCt values of TERT expression, were used (see Section 4.4). For Kaplan-Meier analyses, TERT expression was categorized into two groups using the median expression as a cutpoint. Routine clinical and histopathological categories of breast cancer were applied according to current practice (e.g., ER, PR, HER2 pos vs. neg), as indicated in respective figures and tables. Hardy–Weinberg equilibrium was evaluated by chi-square tests with Yates’ continuity correction. Confidence intervals and *p*-values associated with odds ratios were calculated by the mid-P exact method [38]. Comparisons of continuous variables (e.g., mRNA levels or age at onset) between groups were analyzed with Kruskal-Wallis tests. Follow-up details of our study population, including numbers of events as well as mean and median follow-up times, have been described [42,44]. Survival was analyzed by the Kaplan-Meier method. *p*-values for Kaplan-Meier curves and for cumulative breast cancer incidences were determined by log-rank tests as described [47]. All *p*-values shown are two-sided. Associations with *p*-values < 0.05 were considered statistically significant.

## Figures and Tables

**Table 1:** Association of *TERT* rs10069690 genotypes and alleles with breast cancer risk.

Genotypes/ Alleles	Unadjusted		Adjusted for Age	
	OR (95% CI)	*p*-Value	OR (95% CI)	*p*-Value
TT vs. CC	0.92 (0.51–1.67)	0.793	0.99 (0.51–1.91)	0.957
TT vs. CT	0.93 (0.51–1.69)	0.801	0.95 (0.48–1.89)	0.885
TT vs. CT + CC	0.92 (0.52–1.64)	0.788	0.97 (0.51–1.84)	0.929
CT vs. CC	1.00 (0.72–1.39)	0.990	1.04 (0.72–1.51)	0.833
TT + CT vs. CC	0.99 (0.72–1.35)	0.927	1.03 (0.72–1.47)	0.867
T vs. C	0.98 (0.76–1.25)	0.851	1.01 (0.77–1.34)	0.927

Analyses of breast cancer cases vs. controls of the indicated genotypes or alleles are shown. Analyses were performed unadjusted or adjusted for age as indicated. OR, odds ratio; 95% CI, 95% confidence interval.

**Table 2:** Association of *TERT* SNV rs10069690 with breast cancer risk in patient subpopulations.

	Subgroup	Patient No. (%)	TT vs. CC		T vs. C	
			OR (95% CI)	*p*	OR (95% CI)	*p*
age (years)	<55	171 (42.4%)	1.13 (0.56–2.27)	0.659	1.00 (0.74–1.35)	0.993
	≥55	232 (57.6%)	0.76 (0.38–1.55)	0.425	0.96 (0.72–1.28)	0.775
tumor type	ductal	245 (75.4%)	1.00 (0.52–1.92)	0.868	1.00 (0.76–1.31)	0.977
	lobular	80 (24.6%)	0.61 (0.22–1.71)	0.413	0.73 (0.48–1.10)	0.129
lymph node status	pN0	193 (59.4%)	1.20 (0.61–2.35)	0.548	1.08 (0.81–1.44)	0.618
	pN+	132 (40.6%)	0.48 (0.19–1.25)	0.112	0.78 (0.55–1.11)	0.165
ER status	pos	270 (69.8%)	0.66 (0.33–1.32)	0.257	0.88 (0.66–1.15)	0.345
	neg	117 (30.2%)	1.63 (0.78–3.40)	0.208	1.20 (0.86–1.67)	0.275
PR status	pos	186 (48.9%)	0.64 (0.29–1.39)	0.301	0.84 (0.62–1.14)	0.256
	neg	194 (51.1%)	1.22 (0.62–2.41)	0.546	1.11 (0.83–1.48)	0.485
HER2 status	pos	74 (20.2%)	1.08 (0.43–2.73)	0.723	0.99 (0.66–1.48)	0.960
	neg	293 (79.8%)	0.94 (0.49–1.77)	0.808	1.00 (0.77–1.31)	0.976
Ki67 pos cells	<50%	278 (90.8%)	0.76 (0.39–1.48)	0.449	0.90 (0.69–1.18)	0.445
	≥50%	28 (9.2%)	2.66 (0.84–8.44)	0.110	1.56 (0.88–2.75)	0.133
molecular subtype	luminal	292 (75.8%)	0.73 (0.37–1.41)	0.354	0.89 (0.68–1.17)	0.416
	HER2 type	33 (8.6%)	1.46 (0.45–4.81)	0.414	1.13 (0.65–1.95)	0.673
	triple neg	60 (15.6%)	1.87 (0.75–4.71)	0.155	1.35 (0.88–2.05)	0.167

OR, odds ratios; 95% CI, 95% confidence intervals; *p*, *p*-values; ER, estrogen receptor; PR, progesterone receptor; pos, positive; and neg, negative.

## Data Availability

The data presented in this study are available in the article and the Supplementary Materials.

## Supplementary Materials

The following supporting information can be downloaded at: https://www.mdpi.com/article/10.3390/ijms24031825/s1.

## References

[1] O’Sullivan R.J., Karlseder J. (2010). Telomeres: Protecting chromosomes against genome instability. Nat. Rev. Mol. Cell Biol..

[2] de Lange T. (2009). How Telomeres Solve the End-Protection Problem. Science.

[3] Olovnikov A.M. (1973). A theory of marginotomy. The incomplete copying of template margin in enzymic synthesis of polynucleotides and biological significance of the phenomenon. J. Theor. Biol..

[4] Barthel F.P., Wei W., Tang M., Martinez-Ledesma E., Hu X., Amin S.B., Akdemir K.C., Seth S., Song X., Wang Q. (2017). Systematic analysis of telomere length and somatic alterations in 31 cancer types. Nat. Genet..

[5] Hanahan D., Weinberg R.A. (2011). Hallmarks of cancer: The next generation. Cell.

[6] Blackburn E.H. (2005). Telomeres and telomerase: Their mechanisms of action and the effects of altering their functions. FEBS Lett..

[7] Robinson N.J., Schiemann W.P. (2022). Telomerase in Cancer: Function, Regulation, and Clinical Translation. Cancers.

[8] Collins K., Mitchell J.R. (2002). Telomerase in the human organism. Oncogene.

[9] Greider C.W., Blackburn E.H. (1985). Identification of a specific telomere terminal transferase activity in Tetrahymena extracts. Cell.

[10] Morales C.P., Holt S.E., Ouellette M., Kaur K.J., Yan Y., Wilson K.S., White M.A., Wright W.E., Shay J.W. (1999). Absence of cancer-associated changes in human fibroblasts immortalized with telomerase. Nat. Genet..

[11] Shay J.W., Bacchetti S. (1997). A survey of telomerase activity in human cancer. Eur. J. Cancer.

[12] Zhang A., Zheng C., Lindvall C., Hou M., Ekedahl J., Lewensohn R., Yan Z., Yang X., Henriksson M., Blennow E. (2000). Frequent amplification of the telomerase reverse transcriptase gene in human tumors. Cancer Res..

[13] Blackburn E.H., Greider C.W., Szostak J.W. (2006). Telomeres and telomerase: The path from maize, Tetrahymena and yeast to human cancer and aging. Nat. Med..

[14] Nakamura T.M., Morin G.B., Chapman K.B., Weinrich S.L., Andrews W.H., Lingner J., Harley C.B., Cech T.R. (1997). Telomerase catalytic subunit homologs from fission yeast and human. Science.

[15] Savage S.A., Stewart B.J., Eckert A., Kiley M., Liao J.S., Chanock S.J. (2005). Genetic variation, nucleotide diversity, and linkage disequilibrium in seven telomere stability genes suggest that these genes may be under constraint. Hum. Mutat..

[16] Baird D.M. (2010). Variation at the TERT locus and predisposition for cancer. Expert Rev. Mol. Med..

[17] Bojesen S.E., Pooley K.A., Johnatty S.E., Beesley J., Michailidou K., Tyrer J.P., Edwards S.L., Pickett H.A., Shen H.C., Smart C.E. (2013). Multiple independent variants at the TERT locus are associated with telomere length and risks of breast and ovarian cancer. Nat. Genet..

[18] Haiman C.A., Chen G.K., Vachon C.M., Canzian F., Dunning A., Millikan R.C., Wang X., Ademuyiwa F., Ahmed S., Ambrosone C.B. (2011). A common variant at the TERT-CLPTM1L locus is associated with estrogen receptor-negative breast cancer. Nat. Genet..

[19] Rafnar T., Sulem P., Stacey S.N., Geller F., Gudmundsson J., Sigurdsson A., Jakobsdottir M., Helgadottir H., Thorlacius S., Aben K.K. (2009). Sequence variants at the TERT-CLPTM1L locus associate with many cancer types. Nat. Genet..

[20] Wolpin B.M., Rizzato C., Kraft P., Kooperberg C., Petersen G.M., Wang Z., Arslan A.A., Beane-Freeman L., Bracci P.M., Buring J. (2014). Genome-wide association study identifies multiple susceptibility loci for pancreatic cancer. Nat. Genet..

[21] Huo D., Feng Y., Haddad S., Zheng Y., Yao S., Han Y.J., Ogundiran T.O., Adebamowo C., Ojengbede O., Falusi A.G. (2016). Genome-wide association studies in women of African ancestry identified 3q26.21 as a novel susceptibility locus for oestrogen receptor negative breast cancer. Hum. Mol. Genet..

[22] Michailidou K., Beesley J., Lindstrom S., Canisius S., Dennis J., Lush M.J., Maranian M.J., Bolla M.K., Wang Q., Shah M. (2015). Genome-wide association analysis of more than 120,000 individuals identifies 15 new susceptibility loci for breast cancer. Nat. Genet..

[23] Michailidou K., Lindstrom S., Dennis J., Beesley J., Hui S., Kar S., Lemacon A., Soucy P., Glubb D., Rostamianfar A. (2017). Association analysis identifies 65 new breast cancer risk loci. Nature.

[24] He G., Song T., Zhang Y., Chen X., Xiong W., Chen H., Sun C., Zhao C., Chen Y., Wu H. (2019). TERT rs10069690 polymorphism and cancers risk: A meta-analysis. Mol. Genet. Genom. Med..

[25] Li Z.Y., Dong Y.L., Feng Y., Zhang Z., Cao X.Z. (2016). Polymorphisms in the telomerase reverse transcriptase promoter are associated with risk of breast cancer: A meta-analysis. J. Cancer Res. Ther..

[26] Lilyquist J., Ruddy K.J., Vachon C.M., Couch F.J. (2018). Common Genetic Variation and Breast Cancer Risk-Past, Present, and Future. Cancer Epidemiol. Biomark. Prev..

[27] Killedar A., Stutz M.D., Sobinoff A.P., Tomlinson C.G., Bryan T.M., Beesley J., Chenevix-Trench G., Reddel R.R., Pickett H.A. (2015). A Common Cancer Risk-Associated Allele in the hTERT Locus Encodes a Dominant Negative Inhibitor of Telomerase. PLoS Genet..

[28] Phan L., Jin Y., Zhang H., Qiang W., Shekhtman E., Shao D., Revoe D., Villamarin R., Ivanchenko E., Kimura M. ALFA: Allele Frequency Aggregator. www.ncbi.nlm.nih.gov/snp/docs/gsr/alfa/.

[29] Shay J.W. (2016). Role of Telomeres and Telomerase in Aging and Cancer. Cancer Discov..

[30] Lacroix M., Leclercq G. (2004). Relevance of breast cancer cell lines as models for breast tumours: An update. Breast Cancer Res. Treat..

[31] Neve R.M., Chin K., Fridlyand J., Yeh J., Baehner F.L., Fevr T., Clark L., Bayani N., Coppe J.P., Tong F. (2006). A collection of breast cancer cell lines for the study of functionally distinct cancer subtypes. Cancer Cell.

[32] Dratwa M., Wysoczanska B., Lacina P., Kubik T., Bogunia-Kubik K. (2020). TERT-Regulation and Roles in Cancer Formation. Front. Immunol..

[33] Darlix A., Louvel G., Fraisse J., Jacot W., Brain E., Debled M., Mouret-Reynier M.A., Goncalves A., Dalenc F., Delaloge S. (2019). Impact of breast cancer molecular subtypes on the incidence, kinetics and prognosis of central nervous system metastases in a large multicentre real-life cohort. Br. J. Cancer.

[34] Wang K., Wang R.L., Liu J.J., Zhou J., Li X., Hu W.W., Jiang W.J., Hao N.B. (2018). The prognostic significance of hTERT overexpression in cancers: A systematic review and meta-analysis. Medicine.

[35] Shay J.W., Wright W.E. (2019). Telomeres and telomerase: Three decades of progress. Nat. Rev. Genet..

[36] Dratwa M., Wysoczanska B., Brankiewicz W., Stachowicz-Suhs M., Wietrzyk J., Matkowski R., Ekiert M., Szelachowska J., Maciejczyk A., Szajewski M. (2022). Relationship between Telomere Length, TERT Genetic Variability and TERT, TP53, SP1, MYC Gene Co-Expression in the Clinicopathological Profile of Breast Cancer. Int. J. Mol. Sci..

[37] Vogelstein B., Lane D., Levine A.J. (2000). Surfing the p53 network. Nature.

[38] Bender R., Lange S. (2001). Adjusting for multiple testing—When and how?. J. Clin. Epidemiol..

[39] Stampfer M.R., Bartley J.C. (1988). Human mammary epithelial cells in culture: Differentiation and transformation. Cancer Treat. Res..

[40] Pacher M., Seewald M.J., Mikula M., Oehler S., Mogg M., Vinatzer U., Eger A., Schweifer N., Varecka R., Sommergruber W. (2007). Impact of constitutive IGF1/IGF2 stimulation on the transcriptional program of human breast cancer cells. Carcinogenesis.

[41] Friesenhengst A., Pribitzer-Winner T., Schreiber M. (2014). Association of the G473A polymorphism and expression of lysyl oxidase with breast cancer risk and survival in European women: A hospital-based case-control study. PLoS ONE.

[42] Proestling K., Hebar A., Pruckner N., Marton E., Vinatzer U., Schreiber M. (2012). The Pro Allele of the p53 Codon 72 Polymorphism Is Associated with Decreased Intratumoral Expression of BAX and p21, and Increased Breast Cancer Risk. PLoS ONE.

[43] Taubenschuß E., Marton E., Mogg M., Frech B., Ehart L., Muin D., Schreiber M. (2013). The L10P Polymorphism and Serum Levels of Transforming Growth Factor β1 in Human Breast Cancer. Int. J. Mol. Sci..

[44] Friesenhengst A., Pribitzer-Winner T., Miedl H., Pröstling K., Schreiber M. (2018). Elevated Aromatase (CYP19A1) Expression Is Associated with a Poor Survival of Patients with Estrogen Receptor Positive Breast Cancer. Horm. Cancer.

[45] Miedl H., Dietrich B., Kaserer K., Schreiber M. (2020). The 40bp Indel Polymorphism rs150550023 in the MDM2 Promoter is Associated with Intriguing Shifts in Gene Expression in the p53-MDM2 Regulatory Hub. Cancers.

[46] R Development Core Team (2009). R: A Language and Environment for Statistical Computing.

[47] Harrington D.P., Fleming T.R. (1982). A Class of Rank Test Procedures for Censored Survival-Data. Biometrika.

