# Supplementary material for: Association of the Telomerase Reverse Transcriptase rs10069690 Polymorphism with the Risk, Age at Onset and Prognosis of Triple Negative Breast Cancer
Source: Int J Mol Sci. 2023 Jan 17;24(3):1825. doi: 10.3390/ijms24031825 (PMC9916321; doi:10.3390/ijms24031825)
Supplement: Supplementary file 1 [file ijms-24-01825-s001.zip › ijms-2113538-Supplementary-Figure S1.pdf]

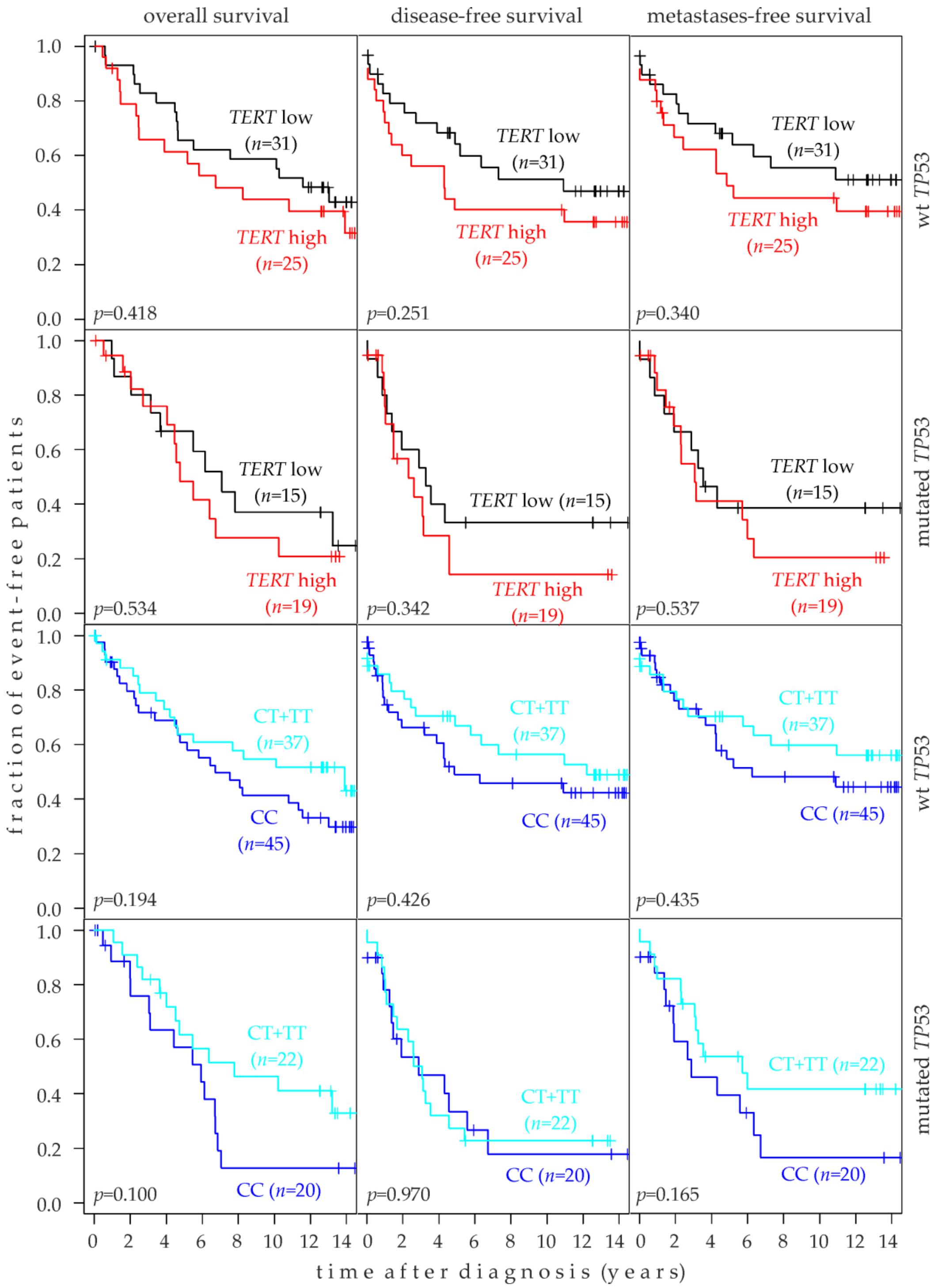

Figure S1. Association of *TERT* mRNA expression and rs10069690 genotypes with the survival of human breast cancer patients with wildtype or mutated *TP53*. Patients with CT and TT genotypes were combined into one group, and compared to CC patients. Kaplan-Meier analyses of the overall survival, disease-free survival and metastasis-free survival in patients with wildtype (wt) *TP53* (top row, *n*=56 and third row, *n*=82), or mutated TP53 (second row; *n*=34 and bottom row, *n*=42) are shown. *TERT* high, *TERT* expression above the median of the study population (*n*=106); *TERT* low, relative *TERT* expression below the median.
