# Supplementary material for: Association of the Telomerase Reverse Transcriptase rs10069690 Polymorphism with the Risk, Age at Onset and Prognosis of Triple Negative Breast Cancer
Source: Int J Mol Sci. 2023 Jan 17;24(3):1825. doi: 10.3390/ijms24031825 (PMC9916321; doi:10.3390/ijms24031825)
Supplement: Supplementary file 1 [file ijms-24-01825-s001.zip › ijms-2113538-Supplementary-Figure S2.pdf]

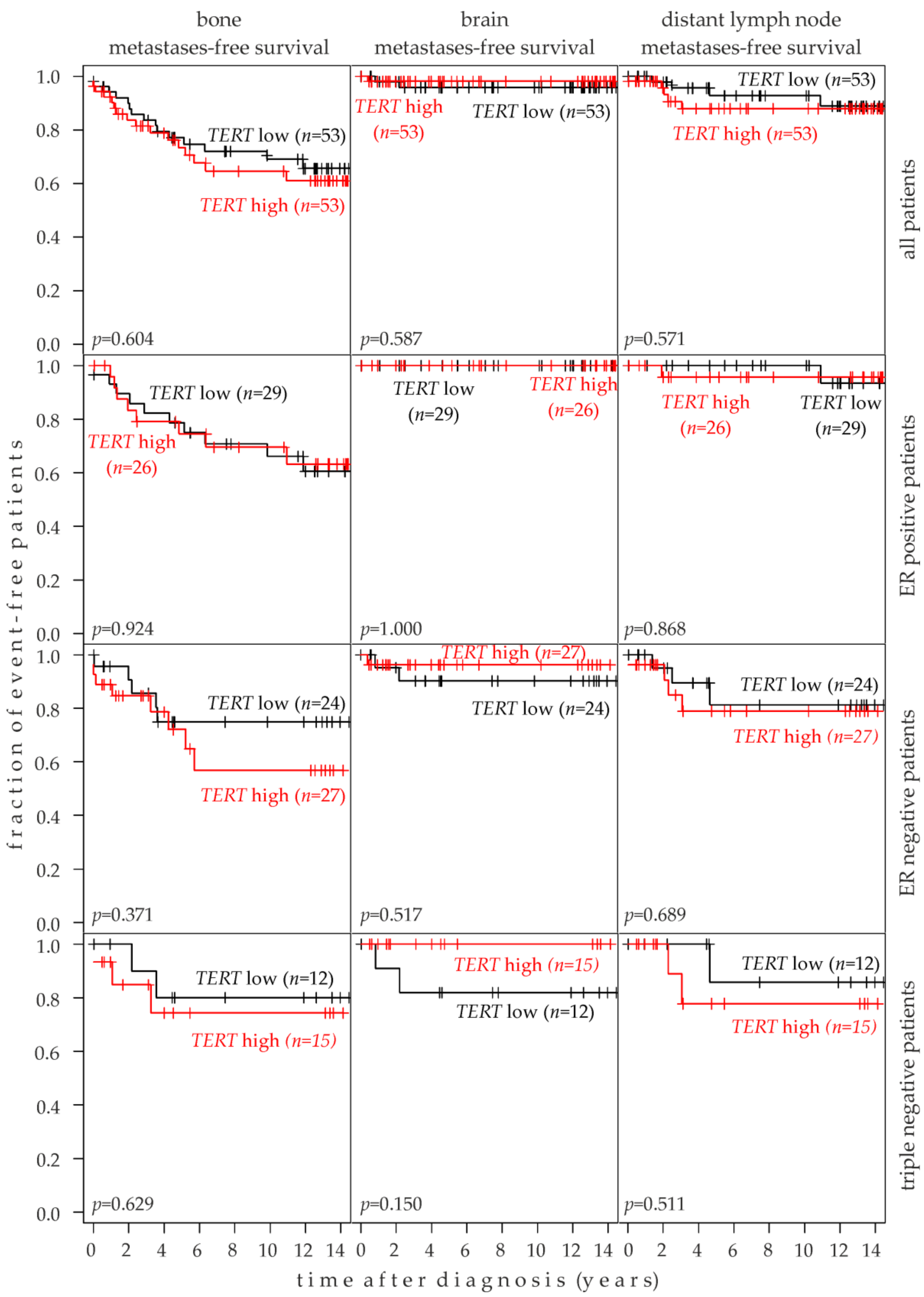

Figure S2. Association of *TERT* mRNA expression with the target tissue specific metastasis-free survival of human breast cancer patients. Kaplan-Meier analyses of the bone metastasis-free survival, brain metastasis-free survival and survival free of metastasis to distant lymph nodes in unselected patients (top row, *n*=106), estrogen receptor (ER) positive patients (second row; *n*=55), ER negative patients (third row; *n*=51), and triple negative patients (bottom row; *n*=27) are shown. Numbers (*n*) of patients in each group and *p*-values (*p*) are indicated. *TERT* high, *TERT* expression above the median of the study population (*n*=106); *TERT* low, relative *TERT* expression below the median.
