# Supplementary material for: Association of the Telomerase Reverse Transcriptase rs10069690 Polymorphism with the Risk, Age at Onset and Prognosis of Triple Negative Breast Cancer
Source: Int J Mol Sci. 2023 Jan 17;24(3):1825. doi: 10.3390/ijms24031825 (PMC9916321; doi:10.3390/ijms24031825)
Supplement: Supplementary file 1 [file ijms-24-01825-s001.zip › ijms-2113538-Supplementary-Table S1.pdf]

Table S1. Clinical characteristics and genotype frequencies of TERT rs10069690 in the study population.

		Sum	CC		CT		TT	
All subjects		649	326	(50.2%)	270	(41.6%)	53	(8.2%)
Patients		403	203	(50.4%)	168	(41.7%)	32	(7.9%)
Controls		246	123	(50.0%)	102	(41.5%)	21	(8.5%)
Patient subpopulations								
Age (years)	<55	171	88	(51.5%)	66	(38.6%)	17	(9.9%)
	≥55	232	115	(49.6%)	102	(44.0%)	15	(6.5%)
Menopausal status	pre	100	54	(54.0%)	36	(36.0%)	10	(10.0%)
	post	248	125	(50.4%)	105	(42.3%)	18	(7.3%)
	na	55	23	(41.8%)	27	(49.1%)	5	(9.1%)
Tumor size	pT1	174	84	(48.3%)	80	(46.0%)	10	(5.7%)
	pT2-4	157	86	(54.8%)	55	(35.0%)	16	(10.2%)
	other, na	73	34	(46.6%)	33	(45.2%)	6	(8.2%)
Tumor type	ductal	245	123	(50.2%)	101	(41.2%)	21	(8.6%)
	lobular	80	48	(60.0%)	27	(33.8%)	5	(6.3%)
	other, na	78	32	(41.0%)	40	(51.3%)	6	(7.7%)
Stage	0-1	141	65	(46.1%)	65	(46.1%)	11	(7.8%)
	2-4	186	103	(55.4%)	67	(36.0%)	16	(8.6%)
	other, na	76	35	(46.1%)	36	(47.4%)	5	(6.6%)
Grade	pG1-2	235	118	(50.2%)	99	(42.1%)	18	(7.7%)
	pG3	145	76	(52.4%)	57	(39.3%)	12	(8.3%)
	na	23	9	(39.1%)	12	(52.2%)	2	(8.7%)
Lymph node status	pN0	193	93	(48.2%)	81	(42.0%)	19	(9.8%)
	pN+	132	73	(55.3%)	53	(40.2%)	6	(4.5%)
	na	72	37	(51.4%)	34	(47.2%)	1	(1.4%)
ER status	pos	270	142	(52.6%)	112	(41.5%)	16	(5.9%)
	neg	117	54	(46.2%)	48	(41.0%)	15	(12.8%)
	na	16	7	(43.8%)	8	(50.0%)	1	(6.3%)
PR status	pos	186	101	(54.3%)	74	(39.8%)	11	(5.9%)
	neg	194	91	(46.9%)	84	(43.3%)	19	(9.8%)
	na	23	11	(47.8%)	10	(43.5%)	2	(8.7%)
HER2 status	pos	74	38	(51.4%)	29	(39.2%)	7	(9.5%)
	neg	293	144	(49.1%)	126	(43.0%)	23	(7.8%)
	na	36	21	(58.3%)	13	(36.1%)	2	(5.6%)
p53 status	pos	91	38	(41.8%)	47	(51.6%)	6	(6.6%)
	neg	257	134	(52.1%)	101	(39.3%)	22	(8.6%)
	na	55	31	(56.4%)	20	(36.4%)	4	(7.3%)
KI67 pos cells	<50%	278	146	(52.5%)	113	(40.6%)	19	(6.8%)
	≥50%	28	11	(39.3%)	12	(42.9%)	5	(17.9%)
	na	97	46	(47.4%)	43	(44.3%)	8	(8.2%)
Tumor Subtype	Luminal A	233	119	(51.1%)	99	(42.5%)	15	(6.4%)
	Luminal B	41	22	(53.7%)	16	(39.0%)	3	(7.3%)
	HER2 type	33	16	(48.5%)	13	(39.4%)	4	(12.1%)
	Triple neg	60	25	(41.7%)	27	(45.0%)	8	(13.3%)
	na	36	21	(58.3%)	13	(36.1%)	2	(5.6%)

Numbers of all patients and controls, as well as number of patients in the indicated subpopulations are shown. Parenthesized numbers show the fraction of patients (%) with the indicated genotypes. ER, estrogen receptor; PR, progesterone receptor; na, status not available.
