# Supplementary material for: Association of the Telomerase Reverse Transcriptase rs10069690 Polymorphism with the Risk, Age at Onset and Prognosis of Triple Negative Breast Cancer
Source: Int J Mol Sci. 2023 Jan 17;24(3):1825. doi: 10.3390/ijms24031825 (PMC9916321; doi:10.3390/ijms24031825)
Supplement: Supplementary file 1 [file ijms-24-01825-s001.zip › ijms-2113538-Supplementary-Table S2.pdf]

Table S2. Pertinent characteristics of the cell lines analysed.

cell line	RRID	cell type	rs10069690 genotype	ER status	*TERT* mRNA levels (linear)	(log[2])
HMEC	CVCL_0307	non-tumor	CT	na	0.78	-0.32
Hs 578Bst	CVCL_0807	non-tumor	CT	na	na	na
MCF-10A	CVCL_0598	non-tumor	CC	neg	71.21	6.19
MCF-10F	CVCL_3633	non-tumor	CC	na	1.22	0.32
AU565	CVCL_1074	tumor	CC	neg	51.45	5,72
BT-474	CVCL_0179	tumor	CT	pos	250.44	8.00
CAL-51	CVCL_1110	tumor	CC	neg	605.29	9.28
CAMA-1	CVCL_1115	tumor	CC	pos	328.29	8.39
HCC1143	CVCL_1245	tumor	CC	neg	2,063.56	11.05
HCC1937	CVCL_0290	tumor	TT	neg	140.78	7.17
Hs 578T	CVCL_0332	tumor	CT	neg	3.46	1.83
KPL-1	CVCL_2094	tumor	CC	pos	1,615.30	10.69
MCF-7	CVCL_0031	tumor	CC	pos	334.19	8.42
MDA-MB-231	CVCL_0062	tumor	CC	neg	1,605.31	10.68
MDA-MB-453	CVCL_0418	tumor	CT	neg	158.61	7.35
MDA-MB-468	CVCL_0419	tumor	CT	neg	82.92	6.41
SK-BR-3	CVCL_0033	tumor	CT	neg	1,007.59	10.01
T-47D	CVCL_0553	tumor	CC	pos	339.36	8.44
ZR-75-1	CVCL_0588	tumor	CC	pos	2,258.41	11.18

Hs 578Bst, MCF-10A and MCF-10F are immortalized cell lines, and HMEC are finite-lifespan cells derived from normal breast tissue. MCF-10A and MCF-10F, as well as Hs 478Bst and Hs578T were derived from the same donor, respectively. *TERT* mRNA levels are expressed relative to the mean level of the two non-tumor cell lines HMEC and MCF-10F. Thus, linear values are equivalent to $2^{-\Delta\Delta Ct}$ values, and log[2] values are equivalent to $-\Delta\Delta Ct$ values. RRID, Research Resource Identifiers available from https://scicrunch.org/resources; ER, estrogen receptor; neg, negative; pos, positive; na, status not available.
